# Advances and challenges of aluminum–sulfur batteries

**DOI:** 10.1038/s42004-022-00693-5

**Published:** 2022-07-04

**Authors:** Matthias Klimpel, Maksym V. Kovalenko, Kostiantyn V. Kravchyk

**Affiliations:** 1 Laboratory of Inorganic Chemistry, Department of Chemistry and Applied Biosciences, ETH Zürich, Vladimir-Prelog-Weg 1, CH-8093 Zürich, Switzerland; 2 Laboratory for Thin Films and Photovoltaics, Empa – Swiss Federal Laboratories for Materials Science and Technology, Überlandstrasse 129, CH-8600 Dübendorf, Switzerland

**Keywords:** Batteries, Batteries, Batteries

## Abstract

The search for cost-effective stationary energy storage systems has led to a surge of reports on novel post-Li-ion batteries composed entirely of earth-abundant chemical elements. Among the plethora of contenders in the ‘beyond lithium’ domain, the aluminum–sulfur (Al–S) batteries have attracted considerable attention in recent years due to their low cost and high theoretical volumetric and gravimetric energy densities (3177 Wh L^−1^ and 1392 Wh kg^−1^). In this work, we offer an overview of historical and present research pursuits in the development of Al–S batteries with particular emphasis on their fundamental problem—the dissolution of polysulfides. We examine both experimental and computational approaches to tailor the chemical interactions between the sulfur host materials and polysulfides, and conclude with our view on research directions that could be pursued further.

## Abstract (web summary)

Aluminum–sulfur batteries have a theoretical energy density comparable to lithium–sulfur batteries, whereas aluminum is the most abundant metal in the Earth’s crust and the least expensive metallic anode material to date. Here, the authors review experimental and computational approaches to tailor the chemical interactions between sulfur host materials and polysulfides in Al-S batteries and point towards promising future research directions.

## Introduction

**Fig. 1:**
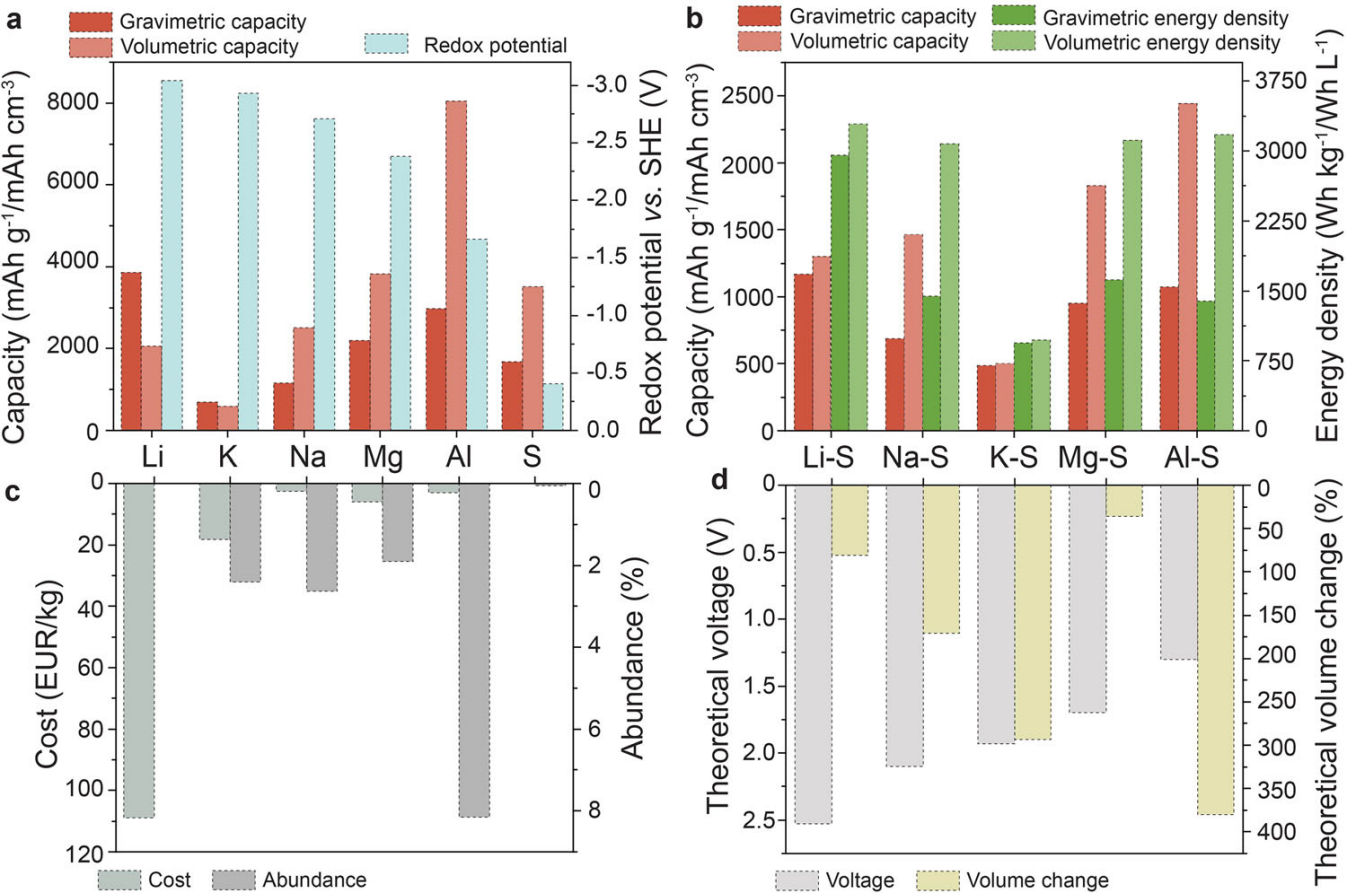
Comparison of Li–S, Na–S, K–S, Mg–S and Al–S batteries and their components. **a** Redox potentials and gravimetric/volumetric capacities of Li, K, Na, Mg, Al and S; **b** Theoretical gravimetric/volumetric capacities and energy densities of Li–S, Na–S, K–S, Mg–S and Al–S batteries; **c** Abundance in Earth crust and cost of Li, K, Na, Mg, Al and S; **d** The theoretical voltage and theoretical volume changes upon cycling of Li–S, Na–S, K–S, Mg–S and Al–S batteries.

Presently, stationary batteries are seen as the ultimate solution to balance rapidly increasing energy consumption with the intermittent nature of renewable energy sources. In the search for sustainable stationary energy storage, over the past decade there have been a number of reports on post-lithium M–S batteries (where M is Na, K, Mg, or Al) as a cost-effective electrochemical technology. In particular, much attention has recently been focused on the development of Al–S batteries, in which aluminum foil can be used as a negative electrode due to the highly reversible and dendrite-free stripping/plating of aluminum^1,2^. Besides, aluminum is the most abundant metal in the earth crust and the least expensive compared to other metallic anode materials (Fig. 1). The redox potential of the Al^3+^/Al redox couple is lower compared to the potentials of the Mg^2+^/Mg, Na^+^/Na and K^+^/K redox couples. However, this is offset by the very high theoretical volumetric capacity of the Al anode (8046 mAh L^−1^). Consequently, resulting theoretical energy density of Al–S batteries on a volume basis equals 3177 Wh L^−1^^3^, similar to that of Na-S batteries (3079 Wh L^−1^)^4^, Mg-S (3115 Wh L^−1^)^5^ as well as Li–S batteries (3290 Wh L^−1^)^6^.

In this review, we provide an introduction to the fundamentals of Al-S batteries and discuss in detail their operating mechanisms. In particular, we examine the factors governing the poor electrochemical performance of the state-of-the-art Al–S batteries, such as the dissolution of Al polysulfides (AlPS) and the slow kinetics of Al_2_Cl_7_^−^ dissociation and Al_2_S_3_ oxidation. We show that the surface functionality of the sulfur host material and/or separator (the polarity, the Lewis acidity) plays an important role in mitigating the issue of polysulfide dissolution. Compared with other reviews published in recent years, e.g., by Sungjemmenla et al.^7^ and Liu et al.^8^, we focus not only on the electrode materials and the deployed electrolyte compositions. We also provide findings on two possible mechanisms of Al–S batteries and discuss theoretical studies on this topic. In addition, we explain common problems that are not directly related to cell performance, such as corrosion of the coin cell casing. To this end, we provide an overview of the prospects of Al–S batteries and emphasize the further research directions and practical hurdles that remain to be explored.

## Historical aspects

**Fig. 2:**
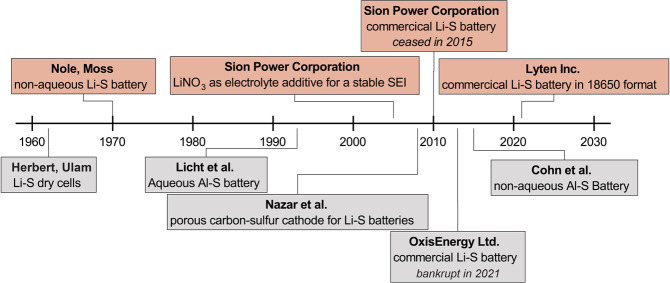
Historical development of Li–S and Al–S batteries. Schematic representation of historical developments in the field of Li–S and Al–S batteries.

The very first idea of employing Metal–S combination for electrochemical energy storage was formulated and patented by Herbert and Ulam in 1962 (Fig. 2)^9^. In particular, they used metallic lithium anode and elemental sulfur cathode, which were combined with an electrolyte based on NaClO_4_ in isoproylamine, saturated with Li^+^ ions. Upon discharge of this battery, the S cathode is reduced to Li_2_S. Concurrently, the lithium anode is oxidized forming Li^+^ ions.

The very first demonstration of the Li–S battery and subsequent experiments have revealed a number of issues associated with this battery concept, such as low cyclic stability, high polarization and low coulombic efficiency. These problems were found to be mainly related to low electronic conductivity of sulfur, formation of polysulfides as reduction intermediates of S and their dissolution in the tested Li-ion electrolytes. In this context, researchers were focusing on solving those issues over the years. The major contribution toward solving these issues was made by the Nazar^10,11^, Manthiram^12,13^ and other groups^14–16^, which facilitated the commercialization of this battery concept by Sion Power Corp^17^, Oxis Energy Ltd^18^, and Lyten Inc^19^. Recently, it was announced that Lyten Inc. has begun production of 18650-format lithium–sulfur batteries for electric vehicles.

**Fig. 3:**
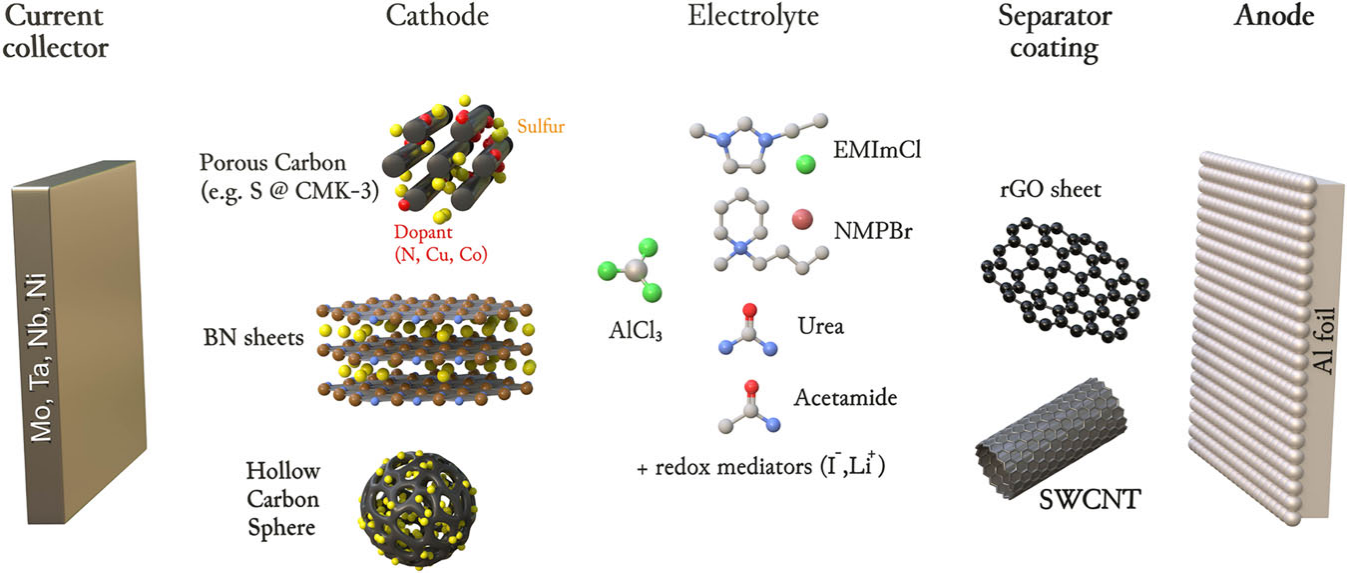
Common materials of state-of-the-art Al-S batteries. The illustration of the common materials used for the fabrication of Al–S batteries.

Investigations of Al-S batteries date back to the 1980s, when Marassi et al.^20,21^ studied sulfur in NaCl-AlCl_3_ electrolyte melts. A second attempt was undertaken in 1993 by Licht and Peramunage using an aqueous alkaline electrolyte^22^. Then, this field of research remained untouched until 2015, when it was proposed to employ non-aqueous ionic liquids as electrolytes for aluminum-sulfur batteries^23^. At that time chloroaluminate ionic liquids were widely used as an Al-ion electrolytes for aluminum plating/stripping with very high efficiency of *ca*. 99.8%^2,24^. On the one hand, the proposed configuration allowed charging of Al–S batteries. On the other hand, the sulfide species were no longer hydrolyzed - the problem that was encountered in aqueous Al–S batteries. Since then, the number of publications on Al–S batteries has been steadily increasing (Supplementary Fig. 1). Figure 3 summaries most common Al-ion electrolytes and materials used in state-of the art Al–S batteries.

## Al-S battery based on chloroaluminate RTIL electrolytes

The configuration of Al-S batteries, commonly reported in publications, is based on chloroaluminate melts, i.e., the mixtures of aluminum chloride and other chlorides containing an organic cation, e.g., 1-butyl-3-methylimidazolium chloride (BMIM) and 1-ethyl-3-methylimidazolium chloride (EMIM)^25^. As a result of the acid-base interactions between AlCl_3_ (Lewis acid) and Cl^−^ (Lewis base), the salt mixture becomes liquid at room temperature (room temperature ionic liquid, RTIL). The latter consists of AlCl_4_^−^ anions whose charge is balanced by organic cations. RTIL with an excess of the Lewis acid AlCl_3_ over the Lewis base EMIMCl consists of both AlCl_4_^−^ and Al_2_Cl_7_^−^ ions.

**Fig. 4:**
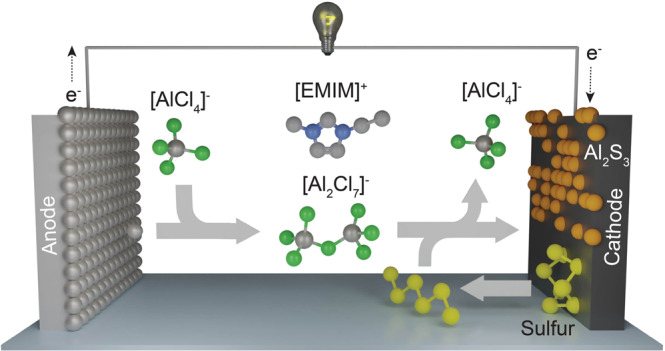
The configuration of an Al–S battery. Schematics of the discharge process in an Al–S battery.

The current understanding of the working mechanism of an Al-S battery comprising chloroaluminate ionic liquid electrolyte can be described by the following half-reactions during discharge and charge (Fig. 4):

1 $${{{{{\rm{Al}}}}}}+7{{AlCl}}_{4}^{-}\leftrightarrow 4{{Al}}_{2}{{Cl}}_{7}^{-}+3{e}^{-}$$

2 $$8{{Al}}_{2}{{Cl}}_{7}^{-}+3{{{{{\rm{S}}}}}}+6{e}^{-}\leftrightarrow {{Al}}_{2}{S}_{3}+14{{AlCl}}_{4}^{-}$$

Although there is no formation of Al^3+^ in the form of solely ions, there is one-directional motion of Al^3+^ in the form of Al_2_Cl_7_^−^ ions, formed upon oxidation of Al in the presence of AlCl_4_^−^ ions. As indicated by Bhauriyal et al.^26^ in a theoretical study using ab initio molecular dynamics, the reaction 2 occurs in 3 steps: (i) S is reduced to S^2−^; (ii) Al_2_Cl_7_^−^ forms AlCl_4_^−^ ions and free Al^3+^ ions; (iii) formed Al^3+^ ions couple with S^2−^, resulting in the formation of Al_2_S_3_.

Importantly, solely Al_2_Cl_7_^−^ ions enable the electroplating of aluminum, which, therefore, occurs only in chloroaluminate melts with an excess of AlCl_3_^27^. The electroplating and thus the charging process ends when there are no more Al_2_Cl_7_^−^ ions in the ionic liquid, which leads to the formation of the neutral melt (AlCl_3_:EMIMCl = 1). Therefore, most experiments on Al-S batteries are performed using slightly acidic formulation to prevent the situation when no Al plaiting would occur due to the absence of Al_2_Cl_7_^−^ ions^3,23,28–35^. Another reason of employment of acidic IL formulation is its reactivity with naturally formed Al_2_O_3_ layer on the surface of Al foil, enabling the cleaning of the Al surface for efficient Al plating/stripping reactions^36^. Another important aspect of choroaluminate melts is their corrosiveness. For example, stainless steel coin-type cells corrode in chloroaluminate melts, requiring the use of corrosion-free battery cases. In addition, unlike Li–ion batteries, where the Al foil is the established current collector, the current collectors at the positive sulfur electrode are still under development. Earth-abundant metals such as iron or aluminum are easily oxidized in aluminum electrolytes at the high voltages of >1.5 V vs. Al^3+^/Al used in positive electrode operation. Therefore, oxidatively stable conductive materials such as molybdenum^3,31,34,37–41^, tantalum^33,35^, niobium^32^ or nickel^25,42–44^ are usually used in Al–S batteries.

First experiments on Al-S batteries comprising chloroaluminate Al-ion electrolytes identified serious issues as to their capacity retention. It has been later realized^3,23,45^ that this issue is associated with dissolution of in situ forming polysulfides in ionic liquid electrolyte similarly to the case observed in Li–S batteries^13,46^. The liquid-phase polysulfides have a tendency to migrate through the separator from the cathode to anode during the operation of metal-sulfur batteries. Like that in Li–S cells, the shuttled polysulfides cause unexpected side reactions with Al metal anode. As a result, the cells show fast self-discharge, low Coulombic efficiency, and fast capacity decay.

Attempting to understand issues of the AlPS, the group of Manthiram performed series of Ultraviolet–visible (UV–Vis) spectroscopy measurements of Al–S batteries in chloroaluminate melts^45^. They identified that only high-order polysulfides, such as S_6_^2−^ and S_8_^2−^, are soluble in the electrolyte. The low-order species were not detected by UV–Vis spectroscopies. This finding was also confirmed by DFT calculations, revealing that the binding energy of polysulfide to the chloroaluminate based electrolytes increases with the molecular weight of polysulfide species^27^. As shown in the work of Bhowmik et al.^47^, polysulfides are coordinated to aluminum ions by replacing one or more chloride ligands in AlCl_4_^–^ and Al_2_Cl_7_^–^. Importantly, it has been also revealed that the subsequent charging of discharged cathode does not result in the formation of elemental S, but rather the formation of S_6_^2−^. Therefore, according to Manthiram et al.^45^, charge storage mechanism of Al-S batteries comprising chloroaluminate electrolytes is associated with redox processes between S_6_^2−^ and S^2−^, rather than between S_8_ and S^2−^, when cycled within 0–2 V *vs*. Al^3+^/Al.

### Redox mediators

Considering the high kinetic barrier for the oxidation of Al_2_S_3_ and AlPS, lowering their overpotentials upon charge and thus increasing the resulting capacity of the S cathode is an important aspect. From this perspective, redox mediators that have been actively studied in the field of Li–S batteries have also been tested for Al–S systems by Nuria’s group^40^. It has been shown that the addition of NaI or LiI as electrolyte additives, whose oxidation potentials are well matched to those of Al_2_S_3_ oxidation, leads to a reduction of the charging voltage (*ca*. 0.23 V with addition of 2.3 wt% redox mediators). The explanation of this affect is related to the following two reactions, taking place upon charge of Al_2_S_3_:

3 $${9I}^{-}\to {3I}_{3}^{-}+6{e}^{-}$$

4 $${{Al}}_{2}{S}_{3}+{3I}_{3}^{-}\to 3/8{S}_{8}+{2{Al}}^{3+}+{9I}^{-}$$

When NaI or LiI redox mediators are added, oxidation of Al_2_S_3_ occurs not electrochemically but chemically by reacting with triiodide (I_3_^−^). In contrast, without mediators, Al_2_S_3_ must donate electrons to the current collector (via conducting carbon additive), which is kinetically less favorable given the insulating nature of Al_2_S_3_.

Another example of employment of redox mediators was demonstrated by Manthiram et al.^45^ It was shown that the addition of lithium triflouromethanesulfonate (LiOTf) or lithium bis(trifluoromethanesulfonyl)imide (LiTFSI) to the chloroaluminate ionic liquid electrolyte can facilitate reduction and oxidation of AlPS through an ion exchange reaction. The coordination of Li to S minimizes the formation of Al = S double bonds upon full discharge of the cell, thus promoting reactivation of sulfide species during charge. Such mediator-ion approach greatly improved the reversibility of Al-S batteries, which delivered the initial capacity of 1000 mAh g^−1^ and at least 60% of this capacity was retained after 50 cycles.

### Other kinetic factors: enhancing Al_2_Cl_7_^−^ dissociation by replacing Cl with Br

To reduce the polarization associated with the kinetically limited dissociation of Al_2_Cl_7_^−^, the introduction of a Br atom into the bridge bond has been proposed. Since a Br atom has similar chemical properties to a Cl atom, but has a lower electronegativity (2.96) and larger covalent radius (120 pm) than a Cl atom (3.16 and 102 pm), a weaker bridge bond (Al−Br) can be expected. The group of Feng showed that an RTIL based on aluminum chloride and 1-ethyl-3-methylimidazoliumbromide improves the dissociation of Al_2_Cl_6_Br^−^ ion^48^. These results were confirmed by DFT calculations showing that the energy barrier for dissociation decreases from 0.934 to 0.863 eV compared with solely chloride-based RTIL.

## Al–S batteries with other electrolytes

In the search for an environmentally sustainable alternative to imidazolium-based RTILs, a new class of ionic liquids, named ionic liquid analogs (ILAs), has recently been used as Al–ion electrolytes for Al–S batteries^42,43^. ILAs can be defined as a mixture of a strongly acidic Lewis metal halide and an oxygen-donating amide ligand, such as urea, which acts as a Lewis base. Similar to the AlCl_3_-EMIMCl system, AlCl_3_-urea is formed by the exothermic reaction between AlCl_3_ and urea according to the following equation:

5 $$2{{AlC}l}_{3}+2{urea}\to {Al}{{Cl}}_{4}^{-}+{[{{AlCl}}_{2}{({urea})}_{2}]}^{+}$$

In addition to AlCl_3_-urea ILAs, several articles focus on the electrochemical performance of Al-S batteries with eutectic AlCl_3_-acetamide (AcAm) mixtures^37–39,43,44^. Considering the low cost of ILAs and their environmental friendliness, Al-S batteries with ILA electrolytes have significant environmental and cost advantages over conventional RTILs. As for the capacity retention of Al-S batteries with ILA, it is similar to that of batteries with RTILs, indicating the same issue of polysulfide dissolution. However, no specific experimental data are available to confirm this assumption.

### Water-in-salt electrolytes

Considering that the air-sensitive and highly corrosive properties of ionic liquid electrolytes are an additional obstacle to the development of safe and cost-effective Al–S batteries, a water-in-salt electrolyte has also been tested recently for Al–S batteries. Although water-based electrolytes inherently exhibit narrow electrochemical stability limited by the electrochemical window (thermodynamically as low as 1.23 V) of water, recent experiments have shown that the operational stability window of water-based electrolytes can be increased up to > 2 V using highly concentrated aqueous solutions of lithium salts containing perfluorinated anions such as bis(trifluoromethylsulfonyl)imide (TFSI). As revealed by molecular dynamics simulations, their higher oxidation stability is associated with the formation of a water-depleted zone at the electrode surface due to the accumulation of bis(trifluoromethanesulfonyl)imide (TFSI) anions^49^. It has been shown that 17 M LiTFSI water-in-salt electrolyte with a small addition of Al(OTF)_3_ and HCl can be used in Al–S batteries offering a high electrochemical stability window of ∼3.0 V and no hydrolysis of AlPS^50^. The advantages in electrochemical performance of Al–S batteries employing this electrolyte over chloroaluminate melts is still to be demonstrated. Reported Al–S battery with water-in-salt electrolyte delivered an initial sulfur capacity of 1410 mA h g^−1^ with a rather low capacity retention of *ca*. 30% after 30 cycles.

## Trapping aluminum polysulfides

Physical entrapment of aluminum polysulfides into porous conductive carbon materials such as meso- and microporous carbons used for molten sulfur infiltration was the main initial strategy to solve the aluminum polysulfide entrapment problem. However, it has been found that simple physical confinement is not sufficient to prevent diffusion and shuttling of AlPS over a long-term cycling, resulting in loss of active S material and degradation of capacity. Due to weak intermolecular interactions, the AlPS diffuse out of the S/C cathode and eventually migrate to the Al anode. As had been shown by first-principles calculations, nonpolar carbon materials exhibit very low binding energies with AlPS which are attributed to Van der Waals forces^27^. From this perspective, considering that AlPSs are intrinsically polar species with the terminal sulfur carrying most of the negative charge, a strong chemical interaction between the sulfur host materials and the dissolved AlPS was considered essential to suppress the diffusion of AlPS and thus achieve long cell lifetime. In the following, we present recent developments in the polar-polar and Lewis acid-base interactions of various sulfur hosts with AlPS.

Recently, a variety of polar host materials have been developed to interact with polar AlPS, including modified carbonaceous materials, functional polymeric materials, and carbon-free materials. For instance, it has been demonstrated that wrapping the separator with rGOs was an effective strategy for improving the capacity retention of Al–S batteries compared with batteries with bare separators^35^. This phenomenon can be explained by the fact that rGOs contain hydroxyl, carboxyl, and ester groups that have been shown to effectively bind S and AlPS. Similar results were obtained using porous single-walled carbon nanotubes (SWCNTs)^45^. Porous carbons doped with heteroatoms represent another class of polar carbonaceous materials that have been shown to effectively suppress AlPS dissolution in Al–S batteries^38^. The incorporation of electronegative N atoms into the carbon lattice results in an asymmetric charge distribution and thus affects net polarity and creates binding sites for AlPS. Considering the same idea of immobilizing the polysulfides through the employment of electronegative atoms, such as nitrogen and therefore inhibiting the dissolution of polysulfides, two-dimensional layered materials such as BN have been also successfully tested as sulfur hosts in Al-S batteries, enabling to achieve high capacity retention of 500 mAh g^−1^ after 300 cycles at a current density of 100 mA g^−1^^30^.

Polymeric materials containing highly electronegative elements have been used in conjunction with carbonaceous materials as one of the other approaches to immobilize AlPS. Incorporation of S into sulfurized polyacrylonitrile (SPAN) polymerized from sulfur and polyacrylonitrile has been demonstrated to be beneficial for capacity maintenance of Al-S batteries. Highly electronegative elements (N, S) located in SPAN have been shown to be effective anchor sites. The polar-polar interaction of Al^3+^ with these electronegative atoms is the key. Such a conjugative structure can react with Al^3+^ ions following the formation of an ion coordination bond between Al^3+^ and the negative locations around the sulfur/nitrogen atoms^29^.

In addition to polar–polar interactions, AlPSs can also bind with sulfur hosts via metal–sulfur bonding, considering that polysulfide anions are soft Lewis bases due to the sulfur lone electron pairs. Therefore, Lewis acidic host materials can interact with AlPSs, trapping them on the host surface. For instance, open coordination metal sites in the MOFs can be seen as soft Lewis acids that can coordinate to polysulfide species thus preventing their detrimental dissolution and shuttle effect^31,34^. These consideration were experimentally confirmed employing ZIF-67 as a sulfur host in Al-S batteries resulting in their improved cycling stability. Moreover, it has been shown that Carbonized-MOF (HKUST-1) consisting of Cu nanoparticles has the capability of immobilizing AlPSs^3^. These assumptions have been also assessed by DFT studies by Pathak et al.^27^, confirming the improved bonding strength between polysulfides and copper atoms (−1.11 to −3.56 eV) compared to the bond between non-functionalized carbon (graphene) and sulfur (−0.76 to −0.88 eV).

## On S/S^4+^ mechanism of Al–S batteries

Contrary to the conventional mechanism of Al-S batteries, where elemental Sulfur is reduced to S^2−^ and then oxidized back to S^0^, a different electrochemistry of Al–S batteries has been recently proposed. It has been suggested and experimentally demonstrated that S can undergo reversible oxidation up to S^4+^ in ILA electrolytes if the upper charging voltage of Al–S battery is extended to 2.4 V *vs*. Al^3+^/Al^42,43,51^. As proposed by Li et al.^51^ the oxidation of sulfur results in the formation of SCl_3_AlCl_4_ species via SCl_3_^+^ intermediates, which can then be reduced back to S at *ca*. 1.8 V. These redox processes can be depicted as follows:

6 $$S+7{Al}{{Cl}}_{4}^{-}\leftrightarrow {{SCl}}_{3}{Al}{{Cl}}_{4}+{3{Al}}_{2}{{Cl}}_{7}^{-}+4{e}^{-}$$

Theoretically, one can expect to get the capacity of S up to 3350 mAh g^−1^ for S/S^4+^ redox process, although much smaller capacity values have been reported (100–150 mAhg^−1^ within 1.8–2.4 V voltage range). Additionally, it has been thought that among other advantages of this Al-S electrochemistry is higher capacity retention, compared to conventional Al-S batteries, as a matter of absence of polysulfide formation – a known problem of S/S^2−^ electrochemistry.

## Open problems and questions

**Fig. 5:**
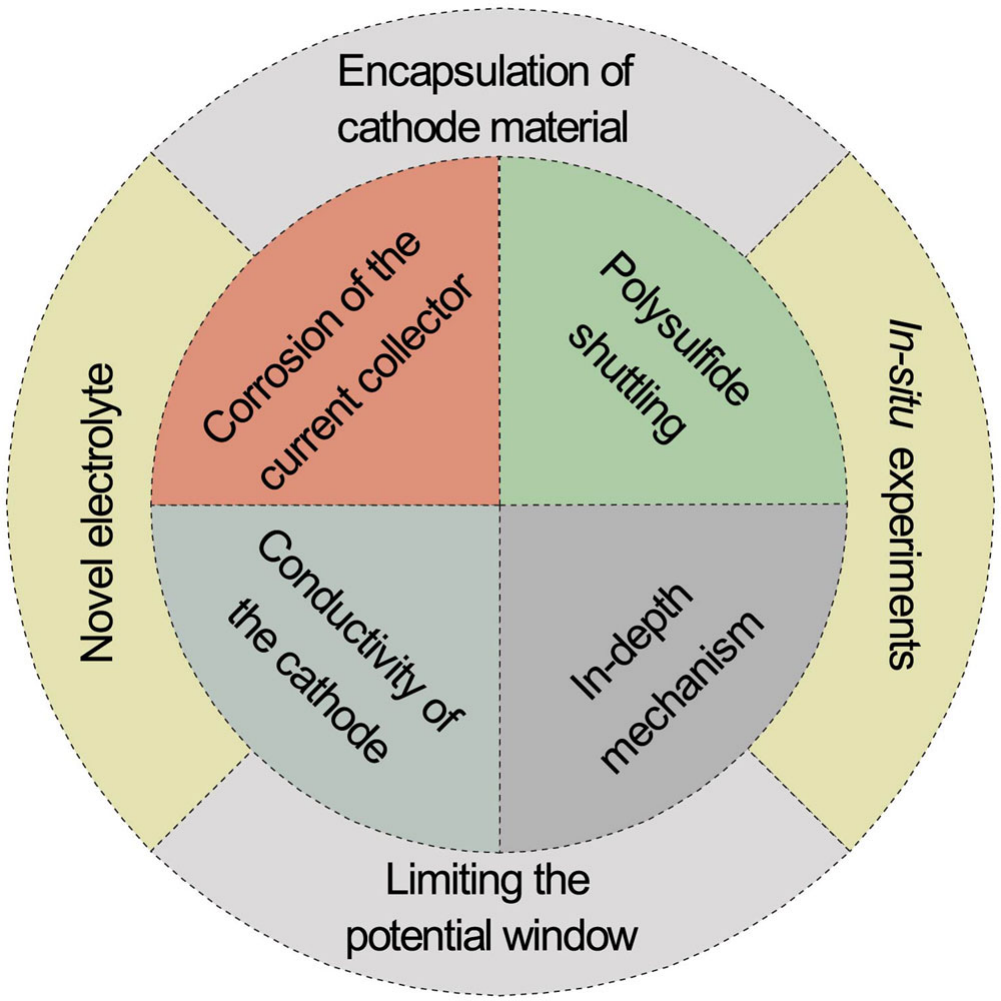
Current issues of Al–S batteries. Summary of the various aspects of Al–S batteries to be addressed.

The field of Al–S batteries has made great strides in understanding the mechanism of sulfur/Al redox reactions, in the advancements of sulfur electrode architecture and Al–ion electrolytes and in the design of functional sulfur-host materials mitigating the issues of the AlPS dissolution. Nevertheless, the development of Al–S batteries is still in its infancy and whether these batteries can eventually be commercialized depends on the solving many aspects of the underlying electrochemistry. They are summarized in Fig. 5 and discussed below. The summary of the reported electrochemical performance of Al–S batteries is shown in Supplementary Fig. 2.

Computation and experiment reports on the mechanism of entrapment of polysulfides demonstrate that the employment of functional sulfur-host materials and separators that bind polysulfides via polar or Lewis acid–base interactions is paramount to ensure high cell capacities and a long cycle life. Apart of the strategy of entrapment of polysulfides, the development of new Al-ion electrolytes with lower polysulfide dissolution can be also considered. The dissolution of AlPSs, like any other Al salts, relies in part on the solvation of Al^3+^ ions. Consequently, the electrolytes with lower electron donor ability (low Gutmann donor numbers) can suppress the solubility of AlPS. From this perspective, it should be possible to decrease polysulfide dissolution via the employment of Cl-free electrolytes^52,53^. In this regards, significant efforts have been made by different group towards the development of Cl-free Al-ion electrolytes. This includes Al(TFSI)_3_ in ACN^54,55^, Al(PF_6_)_3_ in DMSO^56^, aluminum trifluoromethansulfonate (Al(OTF)_3_) in diglyme^57^, N-methyl acetamide/urea^58^, THF^59^ or 1-butyl-3-methylimidazolium trifluoromethanesulfonate^36^. Despite these endeavors, a clear demonstration of efficient Al electrodeposition as well as their employment in Al-S batteries has not yet been demonstrated. It should be also mentioned that other approaches such as employment of polymer Al-ion electrolytes developed by Elia et al.^60^ might be a possible solution in solving the issues of polysulfide dissolution and shuttling. However, it should be clear that complete mitigation of polysulfide dissolution might impact the kinetics of Al–S battery. Therefore, further studies of all these aspects are required.

Introduction of additives to suppress and anchor the polysulfides is an additional point that needs careful study. The available body of knowledge on Li-S batteries suggest that aryl dithiols such as Biphenyl-4,4’-dithiol can be used as an cathode additive altering the dissolution process, since it reacts with short chain polysulfides, enabling to anchor them onto the cathode structure^61^. Additionally, heterocyclic aromatic compounds with nitrogen heteroatom such as Pyrrole might be tested as electrolyte additive to trap polysulfide dissolution. It has been shown that it is oxidized during the cycling to form a protecting electronically conductive surface layer on top of the sulfur cathode. This layer can absorb the intermediate polysulfide species, forming additional paths for the movement of electrons^62^.

Apart of electrolytes and their additives, more efforts are needed in the context of binder development for S cathodes. DFT calculations showed that lithium polysulfides have a strong affinity to carbonyl groups, present in ketones, esters and amides, and a more loose bond to halogenated functional groups^63^. This leads to the conclusion, that PVDF is not suitable as a binder material for fabrication of S electrode due to the absence of a carbonyl group.

Moreover, a deeper understanding of aluminum polysulfide dissolution is needed, that can be realized through in-situ experiments. So far, the publications on Al-S batteries mostly reported ex-situ studies of the Al-ion electrolyte and the sulfur cathode during cycling. After discharge, it has been determined the presence of all possible sulfur species, i.e. elemental sulfur, S_8_^2−^, S_6_^2−^, S_4_^2−^, S_2_^2−^ and S^2−^. However, polysulfide species with an uneven number of sulfur atom were not detected, which can be explained by their fast dissociation to the other species.

In summary, this technology still requires continued advances on multiple fronts, which should be achieved at a low cost to maintain the overall cost-competitiveness of Al-S batteries. However, given recent achievements in somewhat similar Li-S batteries and ongoing discoveries of battery materials and electrolytes accelerated by machine learning, there is an expectation that Al-S batteries will realize their cost-effective potential and enter large-scale deployment.

## Supplementary information

Supplementary Information

## References

[1] Li Q, Bjerrum NJ (2002). Aluminum as Anode for Energy Storage and Conversion: a Review. J. Power Sources..

[2] Jiang T, Chollier Brym MJ, Dubé G, Lasia A, Brisard GM (2006). Electrodeposition of aluminium from ionic liquids: part i—electrodeposition and surface morphology of aluminium from aluminium chloride (AlCl_3_)–1-ethyl-3-methylimidazolium chloride ([EMIm]Cl) ionic liquids. Surf. Coat. Technol..

[3] Guo Y (2019). Carbonized-MOF as a Sulfur Host for Aluminum–Sulfur Batteries with Enhanced Capacity and Cycling Life. Adv. Funct. Mater..

[4] Cao W, Zhang J, Li H (2020). Batteries with High Theoretical Energy Densities. Energy Storage Mater..

[5] Bieker G, Küpers V, Kolek M, Winter M (2021). Intrinsic Differences and Realistic Perspectives of Lithium-Sulfur and Magnesium-Sulfur Batteries. Commun. Mater..

[6] Zhao M, Li B-Q, Zhang X-Q, Huang J-Q, Zhang Q (2020). A Perspective toward Practical Lithium–Sulfur Batteries. ACS Cent. Sci..

[7] Sungjemmenla SCB, Kumar V (2021). Recent advances in cathode engineering to enable reversible room-temperature aluminium-sulfur batteries. Nanoscale Adv..

[8] Liu X, Li Y, Xu X, Zhou L, Mai L (2021). Rechargeable metal (Li, Na, Mg, Al)-sulfur batteries: materials and advances. J. Energy Chem..

[9] Herbert, D. & Ulam, J. Electric Dry Cells and Storage Batteries. US Patent 3,043,896 (1962).

[10] Ji X, Lee KT, Nazar LF (2009). A Highly Ordered Nanostructured Carbon–Sulphur Cathode for Lithium–Sulphur Batteries. Nat. Mater..

[11] Ji X, Nazar LF (2010). Advances in Li–S batteries. J. Mater. Chem..

[12] Manthiram A, Fu Y, Chung S-H, Zu C, Su Y-S (2014). Rechargeable lithium-sulfur batteries. Chem. Rev..

[13] Manthiram A, Fu Y, Su Y-S (2013). Challenges and prospects of lithium-sulfur batteries. Acc. Chem. Res..

[14] Zheng G, Yang Y, Cha JJ, Hong SS, Cui Y (2011). Hollow carbon nanofiber-encapsulated sulfur cathodes for high specific capacity rechargeable lithium batteries. Nano Lett..

[15] Yuan Z (2014). Hierarchical free-standing carbon-nanotube paper electrodes with ultrahigh sulfur-loading for lithium–sulfur batteries. Adv. Funct. Mater..

[16] Urbonaite S, Poux T, Novák P (2015). Progress towards commercially viable li–s battery cells. Adv. Energy Mater..

[17] *Sion Power Corporation*, https://sionpower.com (2021).

[18] *Oxis Energy Ltd*, https://oxisenergy.com/ (2021).

[19] *Lyten Inc*., https://lyten.com/ (2021).

[20] Tanemoto K (1982). Oxidation of sulfur in chloroaluminate melts of intermediate pCl. J. Electrochem. Soc..

[21] Marassi R, Laher TM, Trimble DS, Mamantov G (1985). Electrochemical and spectroscopic studies of sulfur in aluminum chloride‐N‐(n‐Butyl)pyridinium chloride. J. Electrochem. Soc..

[22] Licht S, Peramunage D (1993). Novel aqueous aluminum/sulfur batteries. J. Electrochem. Soc..

[23] Cohn G, Ma L, Archer LA (2015). A novel non-aqueous aluminum sulfur battery. J. Power Sources..

[24] Zhao Y, VanderNoot TJ (1997). Electrodeposition of aluminium from nonaqueous organic electrolytic systems and room temperature molten salts. Electrochim. Acta..

[25] Xia S, Zhang X-M, Huang K, Chen Y-L, Wu Y-T (2015). Ionic liquid electrolytes for aluminium secondary battery: influence of organic solvents. J. Electroanal. Chem..

[26] Bhauriyal P, Das S, Pathak B (2020). Theoretical insights into the charge and discharge processes in aluminum–sulfur batteries. J. Phys. Chem. C..

[27] Bhauriyal P, Pathak B (2020). Superior anchoring effect of a Cu-benzenehexathial MOF as an. Alum.–Sulfur Battery Cathode Host. Mater. Adv..

[28] Gao T (2016). A rechargeable al/s battery with an ionic-liquid electrolyte. Angew. Chem. Int. Ed..

[29] Wang W (2018). Recognizing the mechanism of sulfurized polyacrylonitrile cathode materials for Li–S batteries and beyond in Al–S Batteries. ACS Energy Lett..

[30] Zhang K (2019). Two-dimensional boron nitride as a sulfur fixer for high performance rechargeable aluminum-sulfur batteries. Sci. Rep..

[31] Guo Y (2020). Rechargeable Aluminium–Sulfur Battery with Improved Electrochemical Performance by Cobalt-Containing Electrocatalyst. Angew. Chem. Int. Ed..

[32] Smajic J (2020). Capacity Retention Analysis in Aluminum-Sulfur Batteries. ACS Appl. Energy Mater..

[33] Zhang Y (2021). The Host Hollow Carbon Nanospheres as Cathode Material for Nonaqueous Room-Temperature Al–S Batteries. Int. J. Hydrog. Energy..

[34] Xiao X, Tu J, Huang Z, Jiao S (2021). A Cobalt-Based Metal–Organic Framework and Its Derived Material as Sulfur Hosts for Aluminum–Sulfur Batteries with the Chemical Anchoring Effect. Phys. Chem. Chem. Phys..

[35] Zheng X (2020). Design of a Composite Cathode and a Graphene Coated Separator for a Stable Room-Temperature Aluminum–Sulfur Battery. *Sustain*. Energy Fuels..

[36] Wang H (2016). High-Voltage and Noncorrosive Ionic Liquid Electrolyte Used in Rechargeable Aluminum Battery. ACS Appl. Mater. Interfaces.

[37] Chu W (2019). A Low-Cost Deep Eutectic Solvent Electrolyte for Rechargeable Aluminum-Sulfur Battery. Energy Storage Mater..

[38] Wang J, Xu J, Huang Z, Fan G (2021). Preparation of Nitrogen-Doped Three-Dimensional Hierarchical Porous Carbon/Sulfur Composite Cathodes for High-Performance Aluminum-Sulfur Batteries. Fuller. Nanotub. Carbon Nanostruct..

[39] Zhang D (2021). Highly Reversible Aluminium–Sulfur Batteries Obtained Through Effective Sulfur Confinement with Hierarchical Porous Carbon. J. Mater. Chem. A..

[40] Li H, Lampkin J, Garcia-Araez N (2021). Facilitating Charge Reactions in Al-S Batteries with Redox Mediators. ChemSusChem.

[41] Lampkin J, Li H, Furness L, Raccichini R, Garcia-Araez N (2020). A Critical Evaluation of the Effect of Electrode Thickness and Side Reactions on Electrolytes for Aluminum–Sulfur Batteries. ChemSusChem.

[42] Bian Y (2018). Using an AlCl_3_/Urea Ionic Liquid Analog Electrolyte for Improving the Lifetime of Aluminum-Sulfur Batteries. ChemElectroChem.

[43] Bian Y (2022). Understanding the Oxidation and Reduction Reactions of Sulfur in Rechargeable Aluminum-Sulfur Batteries with Deep Eutectic Solvent and Ionic Liquid Electrolytes. ChemSusChem.

[44] Jiang W, Bian Y, Zhang Y, Lin M (2021). A New Strategy to Improve the Performance of Aluminum-Sulfur Battery. IOP Conf. Ser. Earth Environ. Sci..

[45] Yu X, Boyer MJ, Hwang GS, Manthiram A (2018). Room-Temperature Aluminum-Sulfur Batteries with a Lithium-Ion-Mediated Ionic Liquid Electrolyte. Chem.

[46] Barghamadi M (2014). Lithium–sulfur batteries the solution is in the electrolyte, but is the electrolyte a solution?. Energy Environ. Sci..

[47] Bhowmik A (2022). Influence of ionic coordination on the cathode reaction mechanisms of Al/S batteries. J. Phys. Chem. C..

[48] Yang H (2018). An aluminum–sulfur battery with a fast kinetic response. Angew. Chem. Int. Ed..

[49] Vatamanu J, Borodin O (2017). Ramifications of water-in-salt interfacial structure at charged electrodes for electrolyte electrochemical stability. J. Phys. Chem. Lett..

[50] Hu Z, Guo Y, Jin H, Ji H, Wan L-J (2020). A rechargeable aqueous aluminum–sulfur battery through acid activation in water-in-salt electrolyte. Chem. Commun..

[51] Li H (2021). Reversible electrochemical oxidation of sulfur in ionic liquid for high-voltage Al−S batteries. Nat. Commun..

[52] Mandai T, Johansson P (2016). Haloaluminate-free cationic aluminum complexes: structural characterization and physicochemical properties. J. Phys. Chem. C..

[53] Slim Z, Menke EJ (2022). Aluminum electrodeposition from chloride-rich and chloride-free organic electrolytes. J. Phys. Chem. C..

[54] Krummacher J, Hess LH, Balducci A (2019). Al(TFSI)_3_ in acetonitrile as electrolytes for electrochemical double layer capacitors. J. Electrochem. Soc..

[55] Chiku M, Matsumura S, Takeda H, Higuchi E, Inoue H (2017). Aluminum bis(trifluoromethanesulfonyl)imide as a chloride-free electrolyte for rechargeable aluminum batteries. J. Electrochem. Soc..

[56] Wen X (2021). Synthesis and electrochemical properties of aluminum hexafluorophosphate. J. Phys. Chem. Lett..

[57] Reed LD, Arteaga A, Menke EJ (2015). A combined experimental and computational study of an aluminum triflate/diglyme electrolyte. J. Phys. Chem..

[58] Mandai T, Johansson P (2015). Al conductive haloaluminate-free non-aqueous room-temperature electrolytes. J. Mater. Chem. A..

[59] Slim Z, Menke EJ (2020). Comparing computational predictions and experimental results for aluminum triflate in tetrahydrofuran. J. Phys. Chem..

[60] Elia GA (2021). A gel polymer electrolyte for aluminum batteries. Energy Technol..

[61] Wu H-L, Shin M, Liu Y-M, See KA, Gewirth AA (2017). Thiol-based electrolyte additives for high-performance lithium-sulfur batteries. Nano Energy..

[62] Yang W (2017). Pyrrole as a promising electrolyte additive to trap polysulfides for lithium-sulfur batteries. J. Power Sources..

[63] Seh ZW (2013). Stable cycling of lithium sulfide cathodes through strong affinity with a bifunctional binder. Chem. Sci..

