# Supplementary material for: Advances and challenges of aluminum–sulfur batteries
Source: Commun Chem. 2022 Jul 4;5:77. doi: 10.1038/s42004-022-00693-5 (PMC9814864; doi:10.1038/s42004-022-00693-5)
Supplement: Supplementary file 1 — Supplementary Information [file 42004_2022_693_MOESM1_ESM.docx]

*Supporting Information for*

Advances and Challenges of Aluminum-Sulfur Batteries

*Matthias Klimpel,^1,2^ Maksym V. Kovalenko,^*,1,2^ Kostiantyn V. Kravchyk^*,1,2^*

^1^ Laboratory of Inorganic Chemistry, Department of Chemistry and Applied Biosciences, ETH Zürich, Vladimir-Prelog-Weg 1, CH-8093 Zürich, Switzerland

^2^ Laboratory for Thin Films and Photovoltaics, Empa – Swiss Federal Laboratories for Materials Science and Technology, Überlandstrasse 129, CH-8600 Dübendorf, Switzerland

Corresponding Author

*E-mail: [mvkovalenko@ethz.ch](mailto:mvkovalenko@ethz.ch) and [kravchyk@inorg.chem.ethz.ch](mailto:kravchyk@inorg.chem.ethz.ch)

Keywords: Al-S battery, sulfur, aluminum, stationary energy storage.


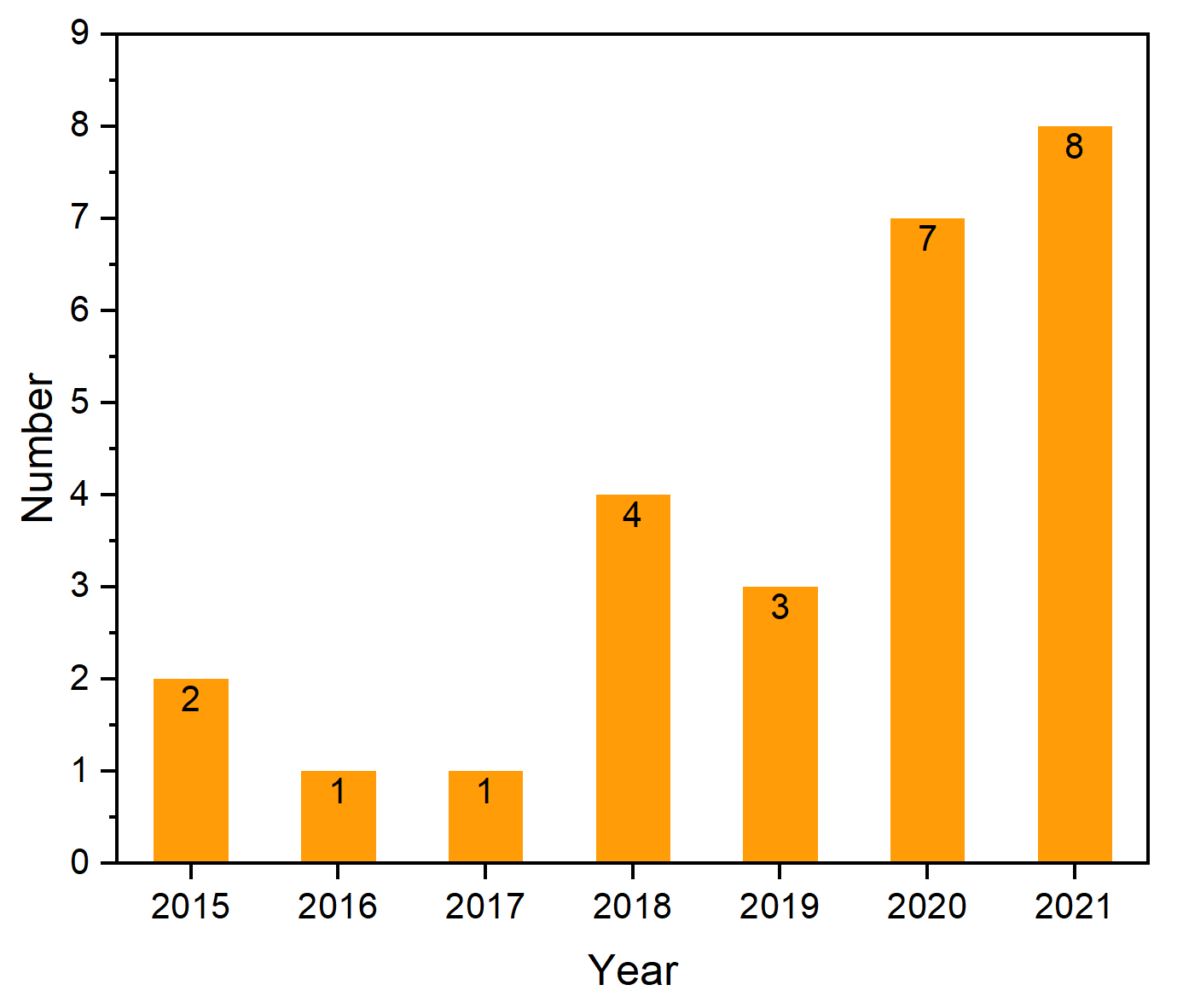


**Supplementary Figure S1.** Numbers of publications on the research topic of aluminum sulfur batteries.


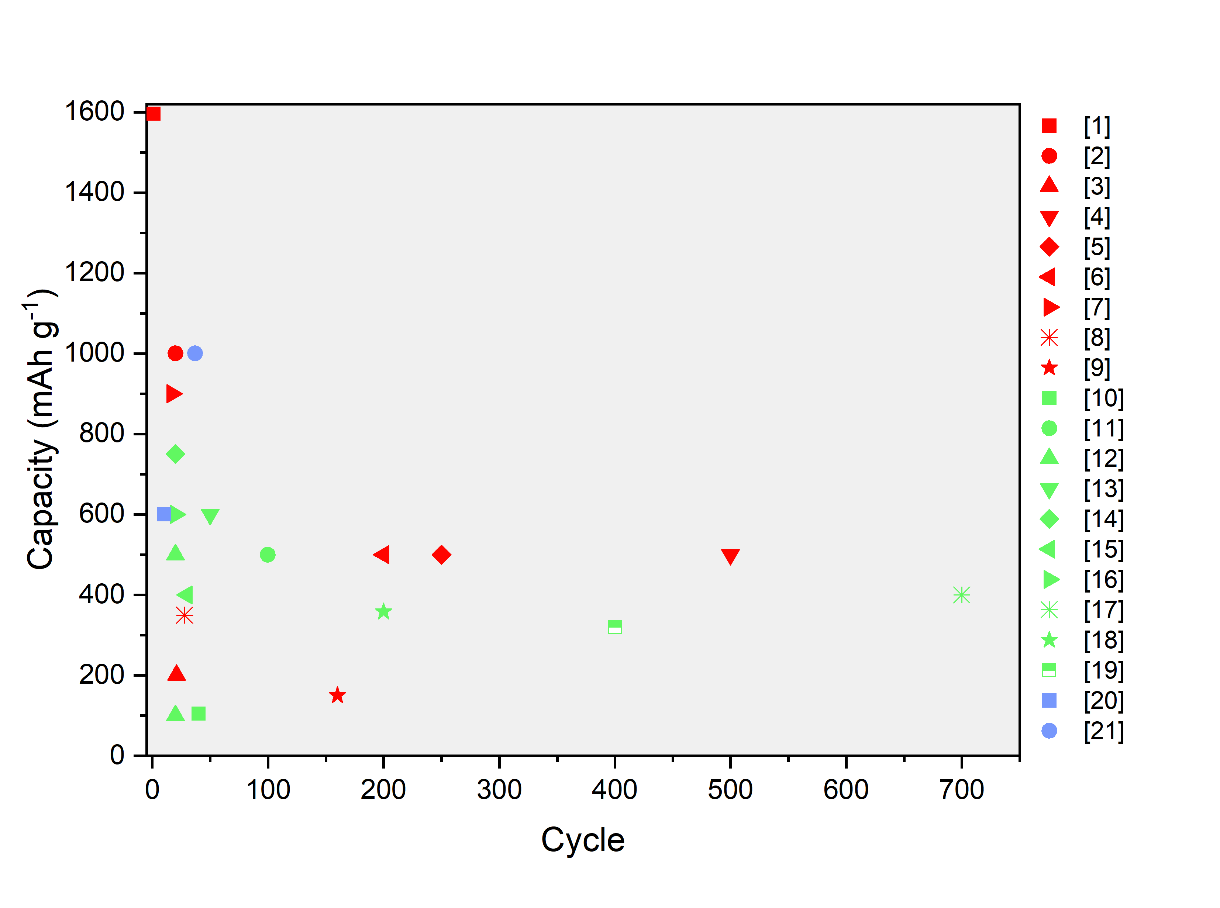


**Supplementary Figure S2.** Performances of aluminum sulfur batteries with the maximum number of cycles and the highest capacity is given.^1-21^ Red dots relate to changes in the carbon host material, green dots correspond to different electrolyte compositions, blue dots show works where the separator was coated.

**Supplementary References**

1. Cohn, G., Ma, L. & Archer, L. A. A novel non-aqueous aluminum sulfur battery. *J. Power Sources* **283**, 416-422 (2015).

2. Gao, T. *et al.* A Rechargeable Al/S Battery with an Ionic-Liquid Electrolyte. *Angew. Chem. Int. Ed.* **55**, 9898-9901 (2016).

3. Wang, W. *et al.* Recognizing the Mechanism of Sulfurized Polyacrylonitrile Cathode Materials for Li–S Batteries and beyond in Al–S Batteries. *ACS Energy Lett.* **3**, 2899-2907 (2018).

4. Guo, Y. *et al.* Carbonized-MOF as a Sulfur Host for Aluminum–Sulfur Batteries with Enhanced Capacity and Cycling Life. *Adv. Funct. Mater.* **29**, 1807676 (2019).

5. Zhang, K. *et al.* Two-dimensional boron nitride as a sulfur fixer for high performance rechargeable aluminum-sulfur batteries. *Sci. Rep.* **9**, 13573 (2019).

6. Guo, Y. *et al.* Rechargeable Aluminium–Sulfur Battery with Improved Electrochemical Performance by Cobalt-Containing Electrocatalyst. *Angew. Chem. Int. Ed.* **59**, 22963-22967 (2020).

7. Smajic, J. *et al.* Capacity Retention Analysis in Aluminum-Sulfur Batteries. *ACS Appl. Energy Mater.* **3**, 6805-6814 (2020).

8. Zhang, Y. *et al.* The host hollow carbon nanospheres as cathode material for nonaqueous room-temperature Al–S batteries. *Int. J. Hydrogen Energy* **46**, 4936-4946 (2021).

9. Xiao, X., Tu, J., Huang, Z. & Jiao, S. A cobalt-based metal–organic framework and its derived material as sulfur hosts for aluminum–sulfur batteries with the chemical anchoring effect. *Phys. Chem. Chem. Phys.* **23**, 10326-10334 (2021).

10. Xia, S., Zhang, X.-M., Huang, K., Chen, Y.-L. & Wu, Y.-T. Ionic liquid electrolytes for aluminium secondary battery: Influence of organic solvents. *J. Electroanal. Chem.* **757**, 167-175 (2015).

11. Bian, Y. *et al.* Using an AlCl_3_/Urea Ionic Liquid Analog Electrolyte for Improving the Lifetime of Aluminum-Sulfur Batteries. *ChemElectroChem* **5**, 3607-3611 (2018).

12. Yang, H. *et al.* An Aluminum–Sulfur Battery with a Fast Kinetic Response. *Angew. Chem. Int. Ed.* **57**, 1898-1902 (2018).

13. Yu, X., Boyer, M. J., Hwang, G. S. & Manthiram, A. Room-Temperature Aluminum-Sulfur Batteries with a Lithium-Ion-Mediated Ionic Liquid Electrolyte. *Chem* **4**, 586-598 (2018).

14. Chu, W. *et al.* A low-cost deep eutectic solvent electrolyte for rechargeable aluminum-sulfur battery. *Energy Storage Mater.* **22**, 418-423 (2019).

15. Hu, Z., Guo, Y., Jin, H., Ji, H. & Wan, L.-J. A rechargeable aqueous aluminum–sulfur battery through acid activation in water-in-salt electrolyte. *Chem. Commun.* **56**, 2023-2026 (2020).

16. Wang, J., Xu, J., Huang, Z. & Fan, G. Preparation of nitrogen-doped three-dimensional hierarchical porous carbon/sulfur composite cathodes for high-performance aluminum-sulfur batteries. *Fuller. Nanotub. Carbon Nanostructures* **29**, 39-45 (2021).

17. Zhang, D. *et al.* Highly reversible aluminium–sulfur batteries obtained through effective sulfur confinement with hierarchical porous carbon. *J. Mater. Chem. A* **9**, 8966-8974 (2021).

18. Jiang, W., Bian, Y., Zhang, Y. & Lin, M. A New Strategy to Improve the Performance of Aluminum-Sulfur Battery. *IOP Conf. Ser. Earth Environ. Sci.* **692**, 032070 (2021).

19. Bian, Y. *et al.* Understanding the Oxidation and Reduction Reactions of Sulfur in Rechargeable Aluminum-Sulfur Batteries with Deep Eutectic Solvent and Ionic Liquid Electrolytes. *ChemSusChem* **n/a** (2021).

20. Ren, Y., Liu, T., Shen, Y., Lin, Y. & Nan, C.-W. Garnet-Type Oxide Electrolyte with Novel Porous-Dense Bilayer Configuration for Rechargeable All-Solid-State Lithium Batteries. *Ionics* **23**, 2521-2527 (2017).

21. Zheng, X. *et al.* Design of a composite cathode and a graphene coated separator for a stable room-temperature aluminum–sulfur battery. *Sustain. Energy Fuels* **4**, 1630-1641 (2020).
